# The Molecular Effects of Dietary Acid Load on Metabolic Disease (The Cellular PasaDoble: The Fast-Paced Dance of pH Regulation)

**DOI:** 10.3389/fmmed.2021.777088

**Published:** 2021-11-16

**Authors:** Morgan Williamson, Naima Moustaid-Moussa, Lauren Gollahon

**Affiliations:** ^1^ Department of Biological Sciences, Texas Tech University, Lubbock, TX, United States; ^2^ Department of Nutrition Sciences, Texas Tech University, Lubbock, TX, United States; ^3^ Obesity Research Institute, Texas Tech University, Lubbock, TX, United States

**Keywords:** diet, pH, obesity, metabolism, homeostasis, disease

## Abstract

Metabolic diseases are becoming more common and more severe in populations adhering to western lifestyle. Since metabolic conditions are highly diet and lifestyle dependent, it is suggested that certain diets are the cause for a wide range of metabolic dysfunctions. Oxidative stress, excess calcium excretion, inflammation, and metabolic acidosis are common features in the origins of most metabolic disease. These primary manifestations of “metabolic syndrome” can lead to insulin resistance, diabetes, obesity, and hypertension. Further complications of the conditions involve kidney disease, cardiovascular disease, osteoporosis, and cancers. Dietary analysis shows that a modern “Western-style” diet may facilitate a disruption in pH homeostasis and drive disease progression through high consumption of exogenous acids. Because so many physiological and cellular functions rely on acid-base reactions and pH equilibrium, prolonged exposure of the body to more acids than can effectively be buffered, by chronic adherence to poor diet, may result in metabolic stress followed by disease. This review addresses relevant molecular pathways in mammalian cells discovered to be sensitive to acid - base equilibria, their cellular effects, and how they can cascade into an organism-level manifestation of Metabolic Syndromes. We will also discuss potential ways to help mitigate this digestive disruption of pH and metabolic homeostasis through dietary change.

## 1 Introduction

### 1.1 Evolution of Dietary pH and Current Public Health Concerns

Diet, along with genetics and environment, affects human health in a plethora of ways. Previous reviews show that some Western style diets may be causative of rising metabolic disease rates (Sebastian et al., 2002; Della Guardia et al., 2016; Della Guardia et al., 2018). Overnutrition and caloric excess with fats and carbohydrates is equally contributory to lower life expectancy as malnutrition (Senior et al., 2020). Most modern diets are thought to contain more meats, animal products, fats, sodium, and chloride, with less fiber and potassium than ancestral diets and even most eastern diets (Frassetto et al., 2001; Sebastian et al., 2002; Cordain et al., 2005; Daniel et al., 2011). Development of transportation, domestication of livestock, industrial revolution, and other advances have allowed our modern diet to overcome food scarcity and other limitations seen in ancestral diet development. Modern diets offer benefits such as ease, convenience, and diversity in food types and flavors. However, they also present drawbacks, examples of which include excess calories and saturated fats, additives, and processed sugars. Current efforts are focused on better understanding how different dietary components affect life expectancy, disease, gut microbiome composition, and other lifestyle dependent changes. Some of these dietary components may be linked to an increase in acid excretion and systemic acidosis underlying these lifestyle-dependent, metabolically-associated diseases.

Metabolic disorders of high concern in today’s society include obesity, insulin resistance and diabetes, chronic inflammation, metabolic acidosis and metabolic syndrome, kidney disease, cardiovascular disease, osteoporosis, and cancers of all kinds. This list encompasses a wide range of disorders that have been linked to each other because they often manifest in combination and share risk factors like poor diet. These diseases also share many symptoms like glucose and hormone imbalance, excess calcium excretion, and oxidative stress. The NIH National Institute of Diabetes and Digestive and Kidney Disease (NIDDK) reported that 70% of the U.S. population is overweight or obese, 15% of the adult population has diabetes, up to 38% have pre-diabetes, and 90% of diabetic patients are also overweight/obese (NIDDK, 2020). Another study shows half of the U.S. population has hypertension (Kirkland et al., 2018). The American Heart Association (AHA) reported 1 in 4 deaths occur from heart disease, about 34% of the population has metabolic syndrome, and an estimated 18% will have chronic or end stage kidney disease (Virani et al., 2021). The same group from the AHA keeps diligent track of statistics for most metabolic diseases with regular updates to this same text. They also emphasize co-morbidities and shared risk factors like blood glucose and cholesterol levels.

Net endogenous acid production (NEAP) has been estimated in previous studies to demonstrate acid load specific to diet composition (Remer and Manz, 1994; Sebastian et al., 2002; Poupin et al., 2012). This calculation also accounts for bicarbonate production potential and any dietary molecules (e.g., Potassium) that may buffer some of the dietary acids. Meat, animal products, and grains are generally net acid producing, whereas vegetables and most fruits contribute to net base production. Animal-derived proteins are a good source of Sulphur-containing amino acids which plant-based foods sometimes lack, making animal protein a potential source of exogenous acids. Fruits and vegetables are a recognized source of complex carbohydrates, fiber, and organic anions precursing base production. Dietary components in a typical US diet, and implicated in metabolic disease, include; excess portion of meats and animal proteins, excess starchy carbohydrates (e.g., refined sugars), and other nutrient poor foods (e.g., processed and commercialized food) (Rush and Yan, 2017). These dietary components amount to a NEAP between 50 and 114 mEq/day on average for Western diet followers (Scialla et al., 2011). Recent studies demonstrate that these trends in food consumption have an impact on pH homeostasis at the cellular level (Baum et al., 2012), which in turns manifests as different metabolic diseases at the organismal level.

## 2 Molecular Regulation of Intracellular pH

pH is defined as −log_10_[H^+^], where H^+^ is the concentration of protons in an aqueous solution.

Free proton concentrations and proton buffering capacities are especially important to the cell. Free protons are exchanged with other ions through the plasma membrane or cellular compartments by various symporters [e.g., Na^+^/HCO_3−_ symporter; major bicarbonate transporter of cells in proximal tubule of kidney (Lee and Hong, 2020)], antiporters [e.g., Cl^−^/HCO_3−_ exchanger; critical for CO_2_ transport in erythrocytes (Lodish et al, 2000)] and channel proteins [e.g., H_v_1; voltage gated proton channel located on plasma membrane of most cell types (DeCoursey and Hosler, 2014)]. Protons can also pass through the cell membrane water channels by piggy-backing onto a chain of water molecules and using the “hop and turn” mechanism (Pomès and Roux, 2002). Protons are more likely sequestered into buffer molecules like HCO_3−_/CO_2_ than freely available. Free protons are only available in the cytoplasm at 10^−7^–10^−8^ M which is low when compared to free potassium ions ([K^+^] = 10^−1^ M) that are less permeable through the cell (Sperelakis, 2012). Maintaining a low free proton concentration is essential to prevent acidosis and activation of pH sensitive processes. pH homeostasis is regulated by many organs and buffer systems that transfer cations and anions through the body. Most significant is HCO_3−_/CO_2_ buffer system (Brinkman JE, 2021). The concentration of HCO_3_^−^ and CO_2_ is monitored and adjusted by the kidneys and lungs to maintain an arterial pH of ∼7.4 (Golebiowski et al., 2021).

**FIGURE 1:**
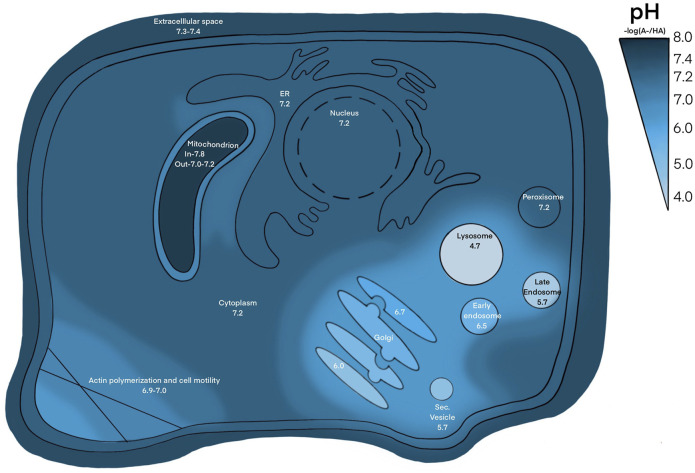
pH through cellular compartments. The pH of extracellular space is ∼ 7.3–7.4, pH of cytosol, nucleus, ER, peroxisome is ∼ 7.2, pH of inner and outer mitochondrial membrane is ∼ 7.8 and 7.0, respectively, pH of golgi ranges from ∼ 6.0–6.7, pH of endosomal and lysosomal compartments range from ∼ 4.5–6.5. Places with high ion trafficking, such as mitochondria-ER interface and cell motility, may have lower pH associated.

**FIGURE 2:**
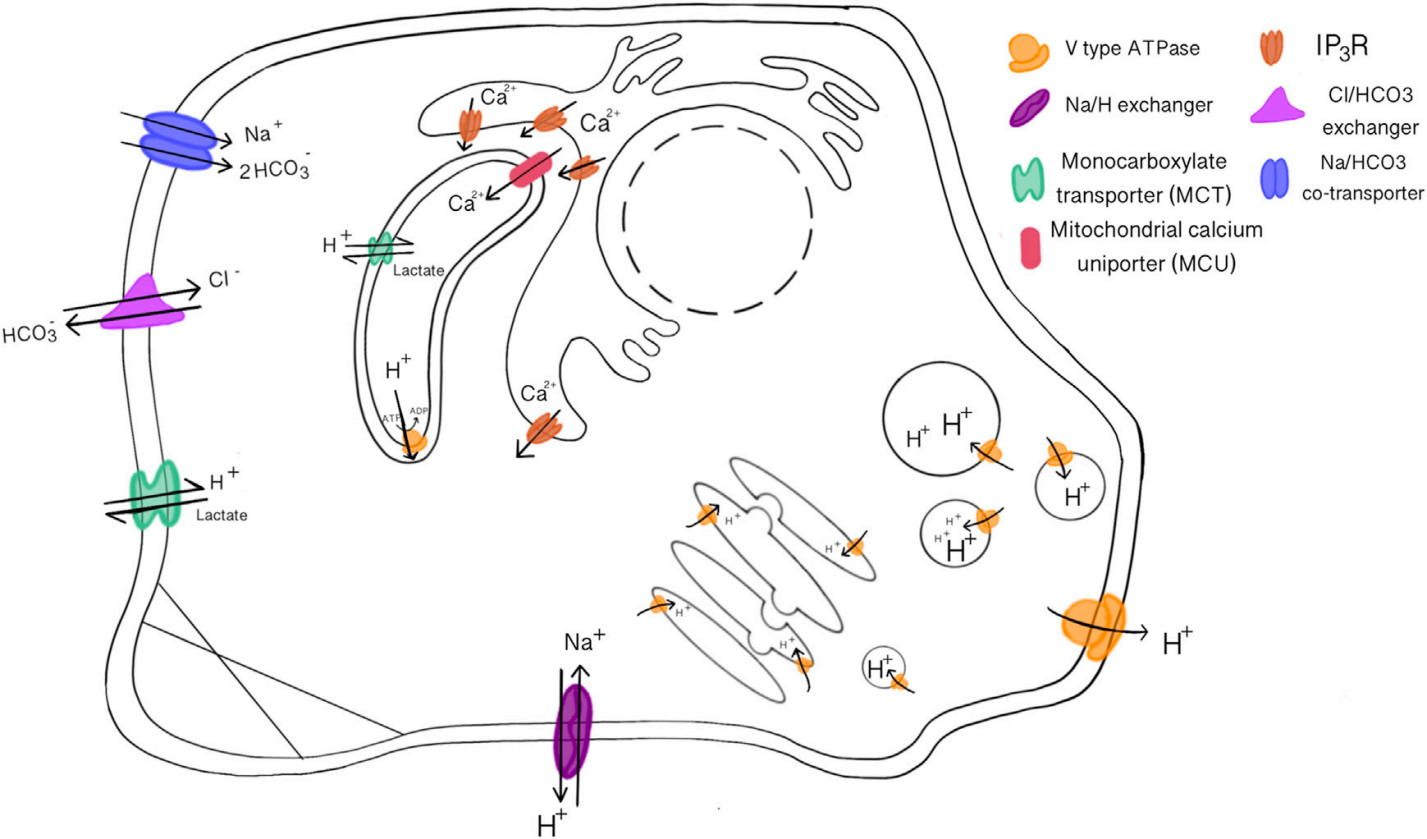
Acid-Base loading/extruding transport proteins that maintain pH homeostasis and membrane potentials. V-type ATPase transport protons across all kinds of membranes, sodium and chloride transport are often coupled with bicarbonate or protons. The mitochondria and ER have increased calcium transporters to execute their functions.

Similar mechanisms are translated to the cellular level, maintaining an extracellular pH between 7.36–7.44 (Goldsmith and Hilton, 1992). Intracellular pH (pH_int_), exemplified in Figure 1, varies through cellular compartments and can change during signal transduction, metabolism, migration, and other events (Goldsmith and Hilton, 1992). The pH_int_ is maintained at around 7.2, regulated by pH sensitive proteins that dictate intricate shuffling of ions through cellular compartments to maintain appropriate buffer concentrations (Zhou et al., 2016). This is further facilitated by various acid loading/extruding and ion exchanging proteins in the membrane (Figure 2). The cytoplasm may also be loaded with acid byproducts from metabolism, leaking of lysosomes or other acidic compartments (Schornack PA, 2003). The nucleus lacks a true pH sensor, but generally equilibrates to the pH_cyt_ of ∼7.2. Most protons enter the nucleoplasm as part of buffer molecules acquired from the cytoplasm (Hulikova and Swietach, 2016). Newer evidence suggests that the nucleus creates a calcium signaling-dependent pH gradient relative to the cytoplasm through proton exchange with Ca^2+^ efflux through nuclear pores (Hulikova and Swietach, 2016). The pH decreases from ER through the golgi to secretory vesicles. The golgi uses a pH gradient to transport vesicles through its stacks, with the cis face at about 6.7 and trans face at ∼6.0. Early endosomes maintain a pH of ∼6.5 and late endosomes are ∼5.4 (Diering and Numata, 2014). Lysosomal compartments are the most acidic at ∼4.7 (Hu et al., 2015). Increasing acidification and dynein on microtubules facilitates endosomal compartment fusion (Hu et al., 2015). Endosomal and lysosomal compartments have V-type ATPases; proton pumps with high affinity to maintain their acidic environments (Diering and Numata, 2014). Unlike other cellular V-ATPases, endosomal V-ATPases do not require co-transport of another ion (Beyenbach and Wieczorek, 2006). Hydrolyses and proteolytic enzymes reside in these compartments and function optimally in these acidic compartments. NHE5 is largely associated with recycling endosomes and neuronal activity through intracellular proton movement into the endosomal lumen (Hu et al., 2015). The mitochondrial matrix is quite alkaline at ∼7.8. The continuous flow of H^+^ into the intermembrane space maintains its slightly acidic pH of ∼7.0. Most notably, proton motive force maintains mitochondrial membrane potential and facilitates ATP production in the inner membrane (Samanta et al., 2018). The ER generally rests at pH ∼7.2 with the cytoplasm, however the ER membrane is highly permeable to H^+^ both passively and through counter transport with calcium (Kim et al., 1998; Li et al., 2020). Other important compartments that may be cell type specific are phagosomes in phagocytic immune cells (Capasso et al., 2011; Levine et al., 2015). The pH values in this section are given as general reference values to facilitate conciseness, please refer to the associated citations which can provide a more complete evaluation of pH range for each compartment.

### 2.1 All Cellular Functions are pH Dependent

The pH is so tightly regulated in the cell, that big effects can be seen with seemingly small pH changes. Fluctuations in intracellular, or intra-compartmental, pH ranging from 0.1–1.6 Units have been documented in various developmental and metabolic functions since the 1980s. It has been suggested that intracellular pH acts as a synergistic messenger for almost all effector molecules in metabolism (Busa and Nuccitelli, 1984). Intracellular pH resists the normal role for a cellular messenger because it does not require any specialized cellular receptors to effect changes. Ion exchange, electrochemical gradients, free energy and transition states in biochemical reactions, and electrostatic interaction, amongst others, rely on protons and acid-base reactions driven by pH and cellular thermodynamics. These mechanisms are the building blocks of cellular functionality and survival.

**FIGURE 3:**
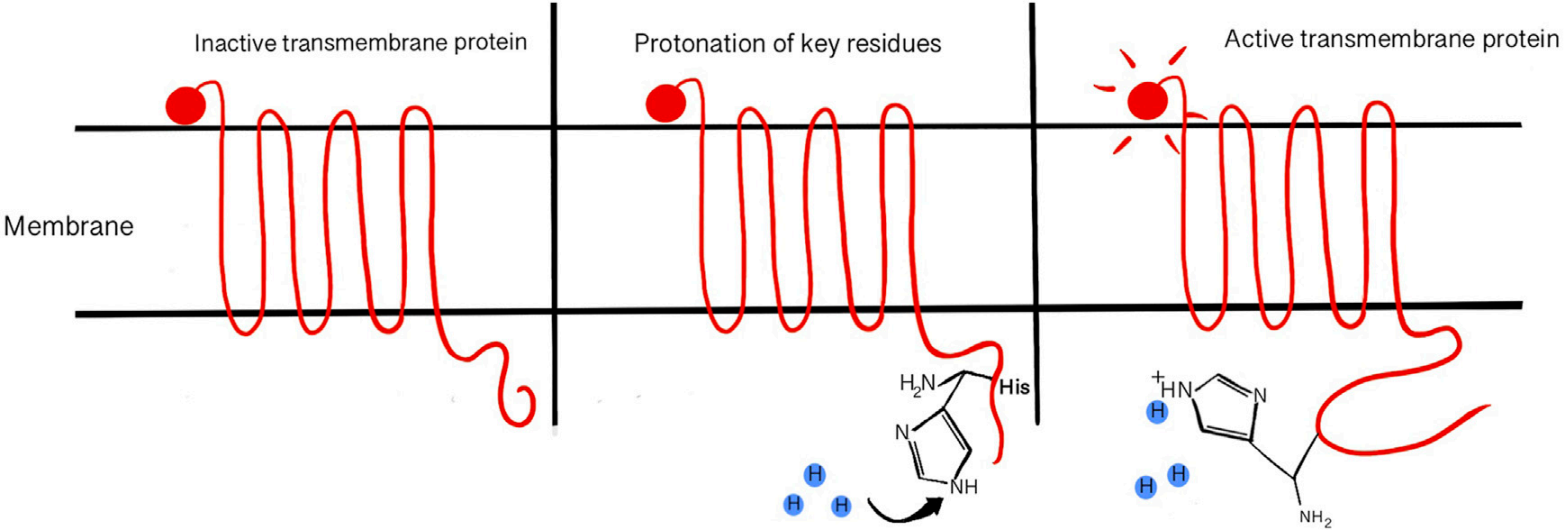
Acid sensing amino acid residues in proteins may become protonated/deprotonated in response to pH changes, causing activating or inhibiting properties. pH can affect the folding structure of a protein.

Protein and DNA formation are pH sensitive, encompassing all cellular events into pH sensitive categories because proteins (which are encoded from DNA) facilitate every cellular activity. Proton concentration can affect binding between nucleotides, backbone components, and melting point for strand separation. Secondary DNA structure is commonly denatured for analysis by molecular techniques like agarose gel electrophoresis that vary the pH. It is also well understood that proteins (of all forms and functions) can be denatured or unfolded from their active forms at nonoptimal pH (Talley and Alexov, 2010). Reducing reagents, like SDS and β-mercaptoethanol, are often used to reduce disulfide bonds in secondary and tertiary structures for protein analysis techniques (Zhao et al., 2018). pH changes affect protein structures on all levels, down to the primary structure which is generally understood to define the rest of the protein shape. Amino acids can have ionizable or titratable groups that allow electrostatic interactions between proximal residues and the environment or associated molecules(Kundrotas and Alexov, 2006; Srivastava et al., 2007). Electrostatic interactions with H^+^ in amino acid residues can cause structural change, inducing activation or other protein activities (McBrian et al., 2013) (Figure 3). The acid dissociation constant (pK_a)_ of amino acids dictates how pH sensitive certain protein motifs can be by estimating their protonation/deprotonation strengths (Lindman et al., 2007; Talley and Alexov, 2010). pK_a_ is determined by the equation, pK_a_ = [H^+^][HA^−^]/[HA]. The pKa of each charged amino acid contributes to the total isoelectric point (pI) of the protein. It was recently found that pI of a protein depends on its localization and is controlled by pH of subcellular compartments (Kurotani et al., 2019). In the currently accepted enzyme-induced fit model, it has been demonstrated that proteins can adjust their conformation to better accommodate their binding partner(s) (Johnson, 2008). Charged amino acids responding to proton concentration may play the biggest role in this phenomenon, allowing the protein to adapt its final structure to each condition (Maiti and Drohat, 2011). The residue(s) responsible for pH sensing and proton binding in relevant proteins can be identified by mutational studies and comparing them to sequence and crystalline structure (Horng et al., 2005; Ishikita, 2011). pH sensitive modifications to protein structure has been reported in protein-protein binding (Sheinerman and Honig, 2002), protein-ligand binding (Onufriev and Alexov, 2013), protein folding and chaperone activity (Simmen et al., 2010), free energy associated with binding and transition states of proteins (Kim and McCammon, 2016), DNA bending, and DNA Base excision repair (BER) (Maiti and Drohat, 2011).

**FIGURE 4:**
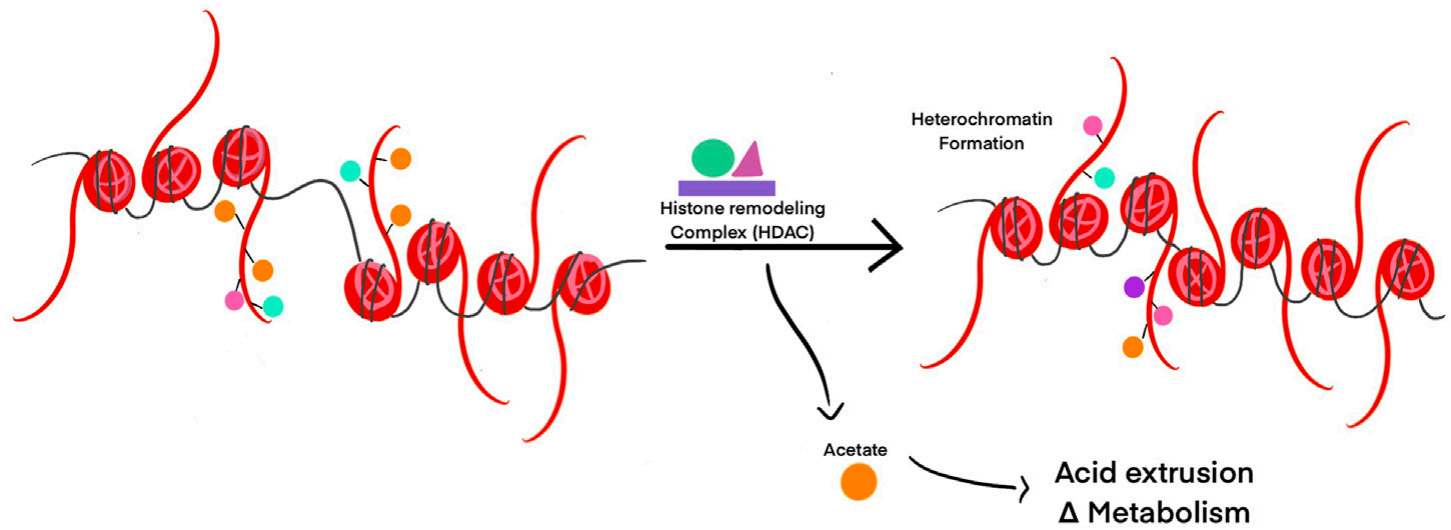
Histone post-translational modifications are modulated by pH changes. HDACs are activated in response to acidic pH to reallocate positively charged ions (such as acetyl groups) and decrease intracellular pH. Removal of acetyl groups on histone tails can lead to heterochromatin formation and transcriptional silencing in those regions of DNA.

Post-translational modifications of proteins such as histones, need a ready source of available charged cellular metabolites like acetyl, methyl, and phosphate groups (Ye and Tu, 2018). Histone remodeling to modulate gene transcription can result in the release of molecules from histone tails. This can directly influence transcription and metabolism by changing the concentration of free metabolites (McBrian et al., 2013). If pH_int_ decreases, histones are deacetylated by HDACs to release acetate ions from the cell along with protons through monocarboxylate transporters to facilitate pH_int_ increase (McBrian et al., 2013) (Figure 4). If pH_int_ increases, histones are increasingly acetylated by HATs to allow transcription for regular cell activity to resume. In this way, intracellular pH controls not only transcription, but replication and cell division as well.

Another important pH dependent cellular event is reduction-oxidation (redox) reactions because most molecules are easily oxidized in the presence of protons (acidic environment) or easily reduced in the presence of hydroxide (basic environment). Redox reactions can result in the formation of reactive oxygen species (ROS) such as hydrogen peroxides, lipid peroxides, and superoxides. ROS often results from normal metabolic pathways and therefore are not entirely avoidable. Some examples include; ER oxidoreductases and chaperone proteins (calnexin and calreticulin) facilitate oxidative protein folding that generates ROS during the formation of disulfide bonds in folding (Simmen et al., 2010). Oxidative metabolism in the mitochondria generates ROS through the electron transport chain (Moxley et al., 2016). Nitric oxide (NO) can be produced through l-arginine metabolism with NOS system (Rajapakse and Mattson, 2009). In response to normal generation of ROS, the cell has adapted many systems involving proteins that sequester ROS or stabilize the radicals [such as; superoxide dismutase (SOD), Glutaredoxin (Grx), glutathione (GSH), and Thioredoxin (Trx)], and antioxidant pathways (e.g., Keap-1-Nrf-2 pathway) (Ray et al., 2012). Understanding antioxidant mechanisms can allow clinicians to evaluate many aspects of health, and more easily diagnose disease, through easy lab tests. Physiological functions, like kidney and erythrocyte, can be measured through detection of ROS or related factors (e.g., SOD, Catylase, and lipid peroxides) in serum or urine (Marrocco et al., 2017). These test results can be indicative of what is happening in the body with respect to pH homeostasis and potentially lead to further insights on treating oxidative stress disorders.

## 3 The Endoplasmic Reticulum Choreographs Other Organelles to Regulate pH Homeostasis

**FIGURE 5:**
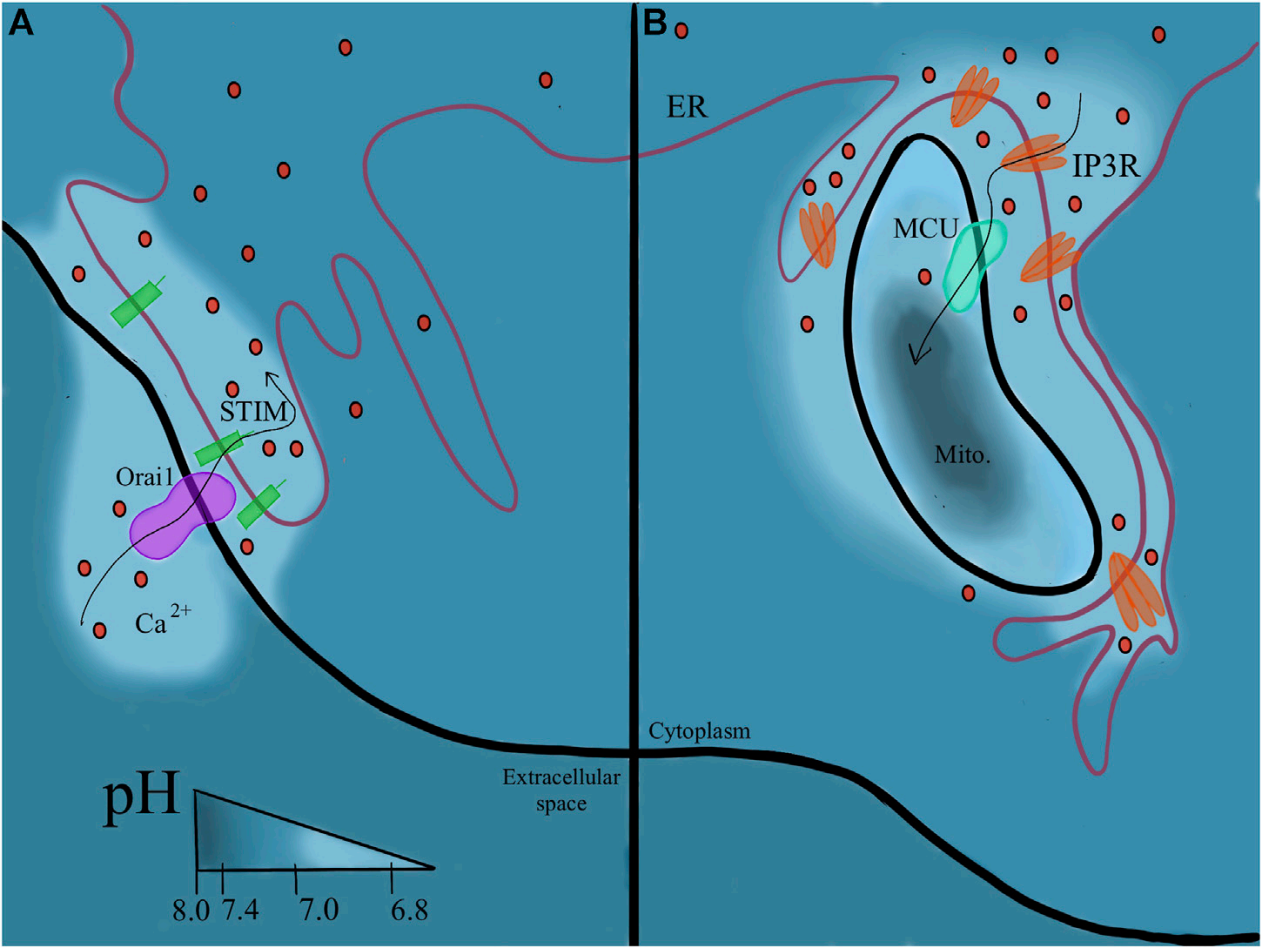
Continuity of the ER membrane orchestrates pH regulation. **(5A)** Plasma membrane associated membrane (PAM). Where the ER contacts the PM, calcium can be trafficked directly into the ER and proton flux follows around these sites. **(5B)** Mitochondrial membrane associated membrane (MAM). pH changes with the flux of calcium ions between the two organelles.

Intracellular and extracellular pH is a factor in an infinite amount of cell regulatory pathways and therefore the cell requires precision from all its organelles to maintain homeostasis. The Endoplasmic Reticulum (ER) is largely responsible for orchestrating the functions of most cellular compartments. The ER is key in regulating lipid metabolism, protein synthesis, protein folding, protein secretion, and storing and managing the cell’s calcium, among other things. It is less well known that the ER, and almost all associated functions, is critical for many aspects of pH homeostasis in the cell. The ER facilitates its functions through its complex shape. As the largest organelle, the ER will take various shapes and distribute itself throughout the cell, through tubule and cisternae formations, while remaining contiguous. Movement of the ER membrane and its proximity (as close as 10–20 nm) with various organelles influences local intracellular pathways by allowing ion fluctuations to be extremely specific in localization and effect (Friedman and Voeltz, 2011). Consequently, these ion movements can be caused by or cause pH changes in the immediate surroundings potentially affecting homeostasis. For example: Movement of calcium is largely dependent on pH and membrane potential (Haumann et al., 2018) (Figure 5). Calcium fluctuations and intracellular pH have been described as interdependent (Busa and Nuccitelli, 1984). pH change around the ER could have an effect on the activity of various ER resident chaperones, modulate protein secretion, lipid homeostasis, and/or receptor-ligand affinity (Simmen et al., 2010).

The enigma (Camello et al., 2002; Lee and Zhao, 2019) of calcium signaling and its extremely important role in all aspects of human health is a highly reviewed topic. To have such a wide range of effects, calcium signaling impacts different pathways by modulating calcium concentration spatially and temporally (Berridge, 2016). The calcium signaling pathways use the unique abilities of the ER to achieve closer proximity and a more specified signal. Store operated Ca^2+^ entry (SOCE) by Orai1 on the plasma membrane, involving STIM1 endoplasmic reticulum calcium sensor (Lin et al., 2019), requires ER proximity to the PM, often called Plasma Membrane-associated membrane (PAM) sites (Lewis, 2007). Orai1 will allow influx of calcium directly into the ER from extracellular space. SOCE is also documented to be extremely pH sensitive (Kimura et al., 2018), suggesting extracellular acidosis can modulate this event. An example is seen in cases of hyperactive SOCE in cancer cells (Mo and Yang, 2018). The continuous extrusion of acid common in cancer cells may increase SOCE. Both extracellular acidosis and increased SOCE are associated with promotion of cancer cell migration and invasiveness (Mo and Yang, 2018) (Figure 5A).

ER contact with the mitochondria allows (mostly) continuous flow of Ca^2+^ ions between the two organelles (Bravo et al., 2011). Thus, increased levels of IP3R are located on Mitochondria-associated membranes (MAM) accordingly. Calcium increases at these sites may contribute to a slightly lower pH (7.0–7.2) at MAM sites and these ions could activate p53, apoptosis, and stress response pathways (Zhou et al., 2020). In addition to calcium signaling, MAM sites are involved in the unfolded protein response (UPR) and translocation of ER-synthesized phospholipids to the mitochondrial membrane (Ma et al., 2017). At MAM sites, chaperone and oxidoreductase interaction dictates the number of MAM sites. This acts as a sensor of sorts for the unfolded protein response. A more complete review of MAMs in cellular homeostasis is provided here (Simmen et al., 2010). The ER also comes into contact with the lysosome to facilitate lipid metabolism (Xu and Huang, 2020) (Figure 5B).

### 3.1 Plasma Membrane-Associated Membranes and Mitochondria-Associated Membranes in Metabolic Disease

Inhibition or dysregulation of the ER membranes’ flexibility can cause or maintain states of disease by loss of control during calcium oscillations (Arruda and Hotamisligil, 2015) and destabilization of cellular membranes (Schuller et al., 2007). Maintenance of cell-wide membrane integrity is dependent on the functional mobility of the ER. Knockout of the regulating protein Opi3, associated with ER-PM contact sites, destabilizes the PM bilayer (Schuller et al., 2007). Increased MAM facilitates stress adaptation of the ER and mitochondria (Bravo et al., 2011). Increased MAM allows an increase calcium uptake which is accompanied by proton influx. Continuous flow of calcium into the mitochondria can cause mitochondrial destabilization and cytochrome C release from the mitochondria to the ER, inhibiting apoptosis. Increase in available protons can drive ATP production and oxygen consumption, causing an increase in ROS production. MAMs are reported to be a site for increased generation of ROS that drives ER stress and activates PKC, this mechanism is implicated in cardiovascular disease (Boengler et al., 2019). Excess protons also drive activity of the proton-dependent receptor responsible for calcium release from ER, IP_3_R (Molinari and Nervo, 2021). Voltage dependent anion channels (VDACs) at MAM sites may be influenced by calcium signaling, pH, and membrane potential and are implicated in neurodegenerative diseases and cancers (Varughese et al., 2021). Stress adaptation by inhibition of apoptosis is often used by cancer cells and observed in cases of metabolic acidosis. Many oncogenes and tumor suppressors, such as p53 and Bcl-XL are located at MAM sites to facilitate apoptotic signaling as a result of the extreme signaling platform MAMs offer (Marchi et al., 2014). MAMs play a role in cardiovascular disease by modulating mitochondrial metabolic dysfunction and more specifically, increasing calcium signaling leading to cardiac hypertrophy and heart failure (Gao et al., 2020). MAM sites are critical to mitochondrial metabolism and cellular adaptation to metabolism with available nutrients. Chronic MAM disruption could inhibit the cells ability to adapt metabolism to nutrient availability, potentially contributing to metabolic disorders (Theurey and Rieusset, 2017). Theurey and Rieusset et al. point to specific nutritional components, like glucose, involved in metabolic adaptation and MAM dysregulation in the molecular origins of Insulin Resistance, Type 2 Diabetes Miletus, and obesity.

## 4 Acidosis and its Association With Molecular Causes of Disease

Much like the age-old predicament of the chicken and the egg, it can be difficult to describe the origins of acidosis and metabolic disease due to cause-and-effect dilemma. Dysregulation of cellular pH and metabolism occurs through signals sent by multiple pathways, often simultaneously. Additionally, modulators of these pathways (ROS, nutrients, ATP, hormones, changes in membrane potential and pH, etc.) rarely appear to act alone and exist at every level from molecular to physiological. This section describes cellular and molecular pathways of metabolic dysfunction and disease with special consideration for the cause/effect of metabolic and cellular acidosis.

It was commonly thought that intracellular pH fluctuations are so quickly buffered that the effect is minimal. However, recent studies have reported that this is not the case. In fact, protons can cause a multitude of effects (promote signaling pathways, activate proteins, stimulate ion exchange, and other activities previously discussed in Section 2) before they are buffered (Molinari and Nervo, 2021). Furthermore, investigations into cellular metabolic kinetics show that metabolic and cellular acidosis can be causative of deregulatory events through chemical/biochemical reaction modulation (Odunewu and Fliegel, 2013; Riemann et al., 2013). These modulations are further facilitated through dietary metabolites and intermediate molecules. Metabolic acidosis (arterial pH > 7.35) lowers intracellular pH slightly to 7.1–7.2, which increases [H^+^] by about 1.0^−8^ mol/L (Salameh et al., 2014). Chronic metabolic acidosis and oxidative stress facilitates intracellular dysregulation through buffering discrepancies (Zhou et al., 2016), redox and metabolism interruption (Moxley et al., 2016), or incorrectly protonated protein residues on important cell signaling receptors (Ishikita, 2011) causing activation of potentially pathological pathways. These dysregulated events are linked to non-communicable diseases such as inflammation (Martinez et al., 2007), insulin resistance (Baldini and Avnet, 2018), development of Type 2 Diabetes Mellitus (Kolb and Martin, 2017), obesity and metabolic syndrome (Frayn, 2001; Rose et al., 2004; Williams et al., 2016), hypertension (Zhang et al., 2009), chronic kidney disease/kidney failure (Welbourne, 1976; Scialla and Anderson, 2013), bone and muscle loss (Sebastian et al., 1994; Frassetto et al., 2018), cancer development and poor prognosis (Fenton and Huang, 2016; Park et al., 2019). These diseases share physiologic characteristics and analogous molecular pathways that are implicated in their pathogenesis, even between cell types. It can be hypothesized that, the conglomeration of multiple cellular stress events, most likely caused by diet induced metabolic acidosis, can contribute to disease progression in any combination of molecular events, contributing to rising occurrences of co-morbidities.

Acid sensing residues in G protein coupled receptors (GPCRs) and Receptor Tyrosine Kinases (RTKs) may be responsible for acidosis-promoted second messenger signaling events that result in immune cell differentiation (Wang et al., 2019), cytokine production (Wang et al., 2019), osteoclast activation (Komarova et al., 2005), ROS generation (Zhou et al., 2016), glucose transport (Sharma and Srikant, 1998), metabolism modulation (Petrenko et al., 2013), cell survival (Petrenko et al., 2013), and oncogenesis (Yu et al., 2018). G protein coupled receptors (GPCRs) can be activated by mildly acidic extracellular conditions (7.0–7.4) and they are widely distributed through cell types (Baltoumas et al., 2013). Acidosis may induce preferential expression of overactive GPCR isoforms with abnormal quantity or location of exposed protein binding motifs in its cytoplasmic domain. An example of this is OGR1 (ovarian cancer G protein coupled receptor 1), among others in the same family, is upregulated in cancers and indicates tumor acidification (Weiss et al., 2017; Abbasalizad Farhangi et al., 2019; Nassios et al., 2019), Certain motifs and the functional shape of the G protein depend on electrostatic interaction between the G protein and the membrane, various isoforms can exist and these dictate the G proteins’ function (Baltoumas et al., 2013). pH around and in the cell influence the functional shape of GPCRs, allowing differential expression and adaptation for various physiologic conditions. This phenomenon may play a role in receptor mediated pathogenesis and the cells’ adaptation to chronic disease signals by GPCRs, such as metabolic acidosis. OGR1, which is pH sensing and proton activated, holds a key role in metabolic acidosis-induced calcium excretion into urine by kidney cells (hypercalciuria) (Abbasalizad Farhangi et al., 2019; Dimke, 2020; Imenez Silva et al., 2020). OGR1 has also been reviewed as a potential drug target (Insel et al., 2020). Metabolic acidosis has been shown to regulate G protein signaling in osteoblasts (Krieger and Bushinsky, 2021).

RTKs, like epidermal growth factor receptor 1 (EGFR), Insulin Receptor (IR), and estrogen receptor (ER), respond to growth factors and hormones e.g., insulin and estrogen, respectively. Some RTKs have been identified as extracellular pH sensing proteins (Zhou et al., 2016). For example, Insulin receptor-related receptor (IRR) monitors pH via [CO_2_/HCO_3−_] in kidney cells to regulate bicarbonate absorption (Petrenko et al., 2013). Similarly, receptor protein tyrosine kinase phosphatase γ plays a functional role in extracellular CO_2_/HCO_3_^−^ sensing in proximal tubule cells of the kidney (Zhou et al., 2016). Both functions are critical for pH homeostasis on the cellular and physiological level. Chronic diet-induced acidosis may hyperactivate or dysregulate these membrane-associated pH sensors. Furthermore, indirect effects of acute or chronic metabolic acidosis, such as increased insulin and cortisol secretion (Baldini and Avnet, 2018), could cause increased activation of RTKs.

Phospholipase C (PLC) and Ras are commonly activated by RTKs in the membrane. PLC activates IP_3_ generation and calcium release from the ER. Protonated IP_3_ is released into the cytosol where it will dissociate from a H^+^ and signal calcium release via the IP_3_R receptor on the ER (Molinari and Nervo, 2021). Moreover, studies show that activation of IP_3_R is proton-dependent, suggesting intracellular acidosis could activate calcium release without external factors or signaling cascade stimulation (Molinari and Nervo, 2021). Ras stimulation can activate p38/MAPK proteins associated with cell cycle progression, cell growth, and various pathologies like cancers (Wagner and Nebreda, 2009). Ras can go on to modulate cell motility, actin and cytoskeletal remodeling, and stimulate cell growth and survival by PI3K/Akt and Rac activation (Cuesta et al., 2021). Ras can also enhance the calcium signaling pathway (Pratt et al., 2020).

These pathways are observed to play a role in acidosis associated increase in cytoplasmic calcium concentrations also seen in obesity related disease (Bagur and Hajnoczky, 2017). The resulting increase in cytosolic calcium will activate many calcium binding proteins such as calcium dependent calmodulin kinase (CaMK), Calmodulin, PKC, and PLA_2_ (Molinari and Nervo, 2021). Calmodulin can activate Calcineurin (calmodulin-dependent serine/threonine phosphatase), allowing the transcription factor NFAT to localize to the nucleus (Park et al., 2020). NFAT activates transcription of genes needed for cytokine production and inflammatory response (Lawrence et al., 2015). Simultaneous activation of pi3K/Akt pathway may cause inhibition of GSK1, a kinase required for NFAT nuclear export, allowing NFAT to remain in the nucleus resulting in chronic inflammation (Komarova et al., 2005). Calcineurin/NFAT pathway activation in non-immune cells is associated with pulmonary hypertension and cardiac hypertrophy (Chou and Chin, 2021), diabetes mellitus and diabetic atherosclerosis (Cai et al., 2021), osteoporosis (Frick et al., 2009), and cancer (Shou et al., 2015). Calcium can activate the inflammatory transcription factor NFkB, both directly and indirectly. NFkB modulates transcription of genes required for immune response (VCAMs, ICAMs, TNFa, COX2) and cell survival (Bcl-Xl, Bcl-2) (Baldini and Avnet, 2018). Extracellular acidosis mediated RANKL stimulation and ROS increase can also activate the NFkB transcription factor (Komarova et al., 2005). Hyperactivation of NFkB inflammatory pathways are associated with insulin resistance, osteoporosis, and cancers (Komarova et al., 2005).

Calcium store depletion will activate SOCE, continuing the cycle of increased calcium signaling by trafficking in more calcium from the extracellular space to replace the stores. GPCRs activate adenylate cyclase (AC) which can stimulate cAMP. PKA activation by cAMP will inhibit SERCA activity, further contributing to sustained high levels of cytoplasmic calcium by inhibiting uptake of calcium into the ER. Increased calcium signaling and associated proton movement may result from increased cytoplasmic calcium and cause increased generation of ROS and increased potential to adapt to stress, among other things previously discussed in Section 3. Urinary analysis of patients with metabolic syndromes reflects metabolic or extracellular acidosis-activated calcium mobilization through increased renal excretion of calcium (Carnauba et al., 2017). This pathway is further evidenced by the requirement of OGR1 (a GPCR), in metabolic acidosis-induced calcium excretion into urine by kidney cells (hypercalciuria) (Abbasalizad Farhangi et al., 2019; Dimke, 2020; Imenez Silva et al., 2020).

Calcium signaling or excess glucose activation of the TCA cycle can increase ROS production (Dejos et al., 2020). Another possibility may be directly related to excess consumption of saturated fats, carbohydrates and processed foods that undergo redox reactions in the digestive tract (Newsholme et al., 2016). Fats are easily oxidized in the gut during digestion by pancreatic lipases (Johnson et al., 2013). Some oxidation products from dietary fatty acids implicated in metabolic disease are reactive aldehydes and ketones such as malondialdehyde (MDA), 4-hydroxy-nonenal (4-HNE), 4-hydroxy-hexenal (4-HHE) and oxysterols (Cisa-Wieczorek and Hernandez-Alvarez, 2020). MDA in particular, is a marker for diabetes due to ROS-induced lipid oxidation (Cisa-Wieczorek and Hernandez-Alvarez, 2020). While the exact molecular mechanisms and dietary origins are still under investigation, these intermediates are implicated in pathogenesis by their ability to modify proteins and DNA via cross-linking, modulate immune responses, bind receptor proteins, and activate NADH oxidase, which generates ROS (Gueraud et al., 2015). Excess ROS and oxidative stress can drive DNA mutagenesis, promoting oncogenesis (Ray et al., 2012). Oxidation of dietary fats and proteins is implicated in the induction of inflammatory markers and colon cancers (Gueraud et al., 2015). Another way diet can induce metabolic stress is by hyperglycemia-induced Nitric Oxide (NO) increase (Macho-Gonzalez et al., 2020). NO, partnered with ROS, further enhances nitrative stress through generation of peroxinitrite (Pivovarova-Ramich et al., 2020). Additionally, excess NO will compete with O_2_ for binding to cytochrome C in the electron transport chain and this nitrative stress is associated with many mitochondrial dysfunction pathologies (Erusalimsky and Moncada, 2007).

### 4.1 Acidosis Drives Activity of Immune Cells

Inflammation is a hallmark of metabolic disease and cancer. Acidosis has been implicated in many cases of pathogenesis by modulating activity in immune cells. Metabolic acidosis activation of the GCPR-Calcium-NFAT pathway may be contributing to the increasing cases of nonallergic respiratory disorders and autoimmune diseases worldwide (Molinari et al., 2020). PLA_2_ and NFAT activation facilitates maturation of granules and subsequent degranulation paired with histamine and other inflammatory cytokine mediators released from the basophils and mast cells (Molinari et al., 2020). Extracellular acidosis promotes maturation of dendritic cells and their production of IL-12 (Martinez et al., 2007). IL-12 promotes differentiation and activation of effector T cells (like Th17), and can activate JAK/STAT cell signaling pathways implicated in inflammatory disease (Gee et al., 2009). Th17 and other T effector cells may prefer glycolysis in a similar way to cancer cells, to produce energy more quickly. T effector cells and cytokine production may also be activated through Pi3K/Akt and/or acid sensing receptor(s) (Nagai et al., 2013). Metabolism generated intracellular ROS drives Th17 differentiation and is required in acute lymphoblastic leukemia (Silva et al., 2011). Hydrogen voltage-gated channel 1 (HVC1) association with BCR is required and helps generate ROS and b cell activity in immune response. HVC1 is upregulated in B cell lymphomas and more reactive to PKC phosphorylation (Hondares et al., 2014). Moreover, metabolic acidosis paired with excess dietary glucose or glucose impairment could further enhance chronic inflammatory symptoms common between many metabolic diseases. Chronic low grade inflammation is sustained in these diseases due to hyperactive phagocytes and favoritism of M1 (pro-inflammatory) macrophages (Olefsky and Glass, 2010). Like T effector cells, M1 macrophages prefer glycolysis for quick energy and high blood glucose levels (from excess consumption and/or glucose impairment) facilitates this kind of metabolism (Zhu et al., 2015). Ultimately, these findings suggest that metabolic acidosis and glucose impairment enhances inflammation associated with autoimmune diseases, allergic-type reactions and infections, metabolic diseases, and cancers.

This overall effect of acidosis on immune cells has repercussions to viral resistance as many viruses also rely on the low pH associated with endocytic vesicles to complete fusion and facilitate uncoating and successful infection. For example, the COVID-19 virus Sars-CoV-2 requires a low pH as well as proteases found in endocytic compartments to induce conformational change of the S (Spike) protein that is responsible for complete fusion and pathogenesis (Belouzard et al., 2012). Metabolic acidosis is a co-morbidity for COVID infection along with chronic kidney disease, diabetes, cardiovascular disease, cancers, and similar metabolism-related pathologies (Ejaz et al., 2020). Among these, hypertension, obesity, and diabetes have the highest fatality rate. Other respiratory viruses, like H1N1 influenza, observe the same co-morbidities (Homsi, 2012). Upregulation of ACE2, documented in these diseases, increases the likelihood of viral binding and entry (Cao et al., 2021). Intracellular increase in proton availability may even facilitate favorable endocytosis and uncoating. Viral RNA, released from successful entry, can trigger formation of NLRP3 inflammasome, which requires mitochondrial destabilization associated with calcium signaling at MAM sites (Horng, 2014). NLRP3 inflammasome formation, calcium signaling, and MAM sites often have increased activity in metabolic diseases, which could contribute to pathogenesis of COVID-19 in patients with co-morbidities. To support these conclusions, the use of calcium channel blockers improves the prognosis of patients with COVID-19 infection as well as hypertension (Peng et al., 2021). Additionally, increased blood glucose enhances COVID-19 infection and is associated with worse prognosis (Sukkar and Bassetti, 2020). SARS-CoV-2 infection causes monocytes and macrophages (prominent and important immune cells in lungs during COVID-19 infection) to switch over to glycolytic metabolism to facilitate viral replication (Jafarzadeh et al., 2020). Glycolysis, and the resulting favor towards M1 macrophage phenotype, is promoted by the stabilization of HIF-1, which is triggered by virally induced mitochondrial ROS generation (Codo et al., 2020).

### 4.2 Acidosis Influences Insulin Secretion and Glucose Transport

ROS generation and oxidative stress are common in metabolic disease pathologies. ROS activates JNK and p38, which activate IRS phosphorylation, causing dissociation of insulin receptor and ubiquitination leading to insulin resistance (Franch et al., 2004). NFAT complexed with p38 and JNK activates transcription of TNF-α (Park et al., 2020). Acidosis and activated NF-kB also enhance this pathway by encouraging stability of the complex at the TNF-α promoter and signaling inflammation (Lawrence et al., 2015). Inhibition of p38 and JNK enhances insulin transcription in pancreatic cells (Lawrence et al., 2015). Insulin stimulates glycolysis and expression of glucose transport proteins (Lodish et al., 2000). The glycolysis product, lactate, is transported out of the cell, coupled with H^+^ efflux through monocarboxylate transporters (MCT), having a net alkalinizing effect on the cytoplasm. Reduced insulin sensitivity or insulin resistance can cause indirect acidosis by preventing lactate metabolism and associated proton extrusion. Chronic intracellular acidosis (pH 7.1) inhibits IRS-1 and Pi3K binding in muscle cells, impairing insulin signaling and insulin’s ability to inhibit proteolysis (Franch et al., 2004). Acidosis stimulates OGR1 activated PLC-calcium signaling, which enhances glucose stimulated insulin secretion and contribute to resistance (Sharma and Srikant, 1998; Rowe et al., 2020). In contrast, cells exposed to chronic hyperinsulinemia will upregulate IR, IRS1, and PI3K activity, while inhibiting Akt phosphorylation, contributing to chronic insulin resistance and high blood sugar seen in obesity and diabetes (Bertacca et al., 2005). Further, hyperinsulemia in early stages of diabetes stimulates SGLT2 (Sodium-coupled glucose transporter 2) upregulation in cells of the proximal tubule (Nakamura et al., 2015). The mechanisms of insulin resistance discussed are encouraged by acidosis and can cause decreased glucose transport into the cell, among other glucose associated impairments (Della Guardia et al., 2018).

Glucose transport is reduced by 39% at pH 7.1 (Teta et al., 2003), which can greatly impact cellular metabolism, signaling, and function. One explanation could be activation of MondoA, a regulator of glucose-dependent transcription, and its preference to bind transcriptional targets that suppress glucose uptake during acidosis (Wilde et al., 2019). Like cancer cells, attempts to normalize cellular pH from acidic conditions cause slowing of glycolysis and associated glucose uptake. This pathway aids cancer cell adaptation, apoptosis evasion, and maintenance of higher intracellular pH to facilitate cancer cell metabolism and progression (Webb et al., 2011). In the same vein, acidosis promotes adaptation to maximize mitochondrial function, energy production, and cell survival, regardless of oxygen availability (Khacho et al., 2014). Khacho et al., demonstrated very distinct changes in mitochondrial morphology that facilitate metabolic adaptation in various conditions simulating oxygen and glucose availability of relevant biological systems.

### 4.3 Intracellular Acidosis Induces Kidney Disease

Metabolic acidosis can be a cause or a result of kidney dysfunction. During chronic metabolic acidosis, the excess intracellular calcium is transported out of the cell and ends up excreted by the renal system due to acidosis-induced inhibition of calcium transporters in the nephron (Alexander et al., 2016). The Bicarbonate buffer system is overwhelmed by the chronic acidosis, causing a decline in glomerular filtration rate (GFR), indicating kidney disease (Golebiowski et al., 2021). Kidney disease is often one of the earliest manifesting consequences of metabolic acidosis. Cells will try to compensate for the acidic environment by excreting acidic molecules into the blood stream for renal excretion. Increased acid excretion by the kidneys lowers urine pH, a key diagnostic factor for metabolic acidosis, kidney disease, and diabetes (Higashiura et al., 2020). Low urine pH is a risk factor for bladder cancer (Wright et al., 2005).

In diabetes, SGLT2 is upregulated in cells of the proximal tubule to increase glucose uptake in response to high blood sugar (Gyimesi et al., 2020). Increased glucose uptake and metabolic acidosis drive gluconeogenesis in these cells in the early stages of diabetes and contributes to metabolic stress. Glucose and sodium transport are generally coupled, causing increased reabsorption of sodium and upregulation of sodium transporters, like NHE3. The increase in reabsorption and filtration is identified by high GFR seen in patients during the onset of diabetes (Nespoux and Vallon, 2020). Hyperabsorption and hyperfiltration can promote inflammation, tissue damage, and eventually declining GFR and fibrosis. Other consequences of hyperfiltration include high blood pressure and cardiovascular disease (Vallon and Thomson, 2020). Metabolic acidosis causes dysregulation of glutamine metabolism. Acidosis shift metabolism of glutamine by kidney cells towards ammoniagenesis and gluconeogenesis by multiple pH sensitive binding sites on promoter of snat3 (Lister et al., 2018). Ammoniagenesis further contributes to acidosis by increases net endogenous acid production and decreasing pH in urine.

### 4.4 Acidosis Increases Intracellular Calcium in Muscle and Bone Cells

Acidosis may encourage proton dependent inhibition of mitochondrial calcium uptake (MCU) proteins in myocytes (Fernandez-Morales and Morad, 2018), contributing to increased calcium in the cytoplasm leading to inflammation and oxidative stress. Similarly, Intracellular acidosis activates NHE1 (Sodium/Proton exchanger isoform 1) through proton interaction with inner transport domain causing phosphorylation of residues on the cytosolic tail (Odunewu and Fliegel, 2013; Lee and Jung, 2017). NHE1 activates PKC, ERK1/2, and p90RSK pathways, and is associated with increased intracellular calcium causing reduced contractability in myocytes (Wu and Kraut, 2014). Obesity has been reported to cause cell calcium dysregulation and intracellular calcium increase, allowing vascular impairment and cardiovascular disease (Wilson et al., 2020). Acidosis causes myocyte death by autophagy, mediated by calcium binding proteins in the BNIP3 pathway (Graham et al., 2015). Increased intracellular calcium contributes to cardiac stress, CVD, and muscle deterioration. These calcium dependent mechanisms are often facilitated by dysregulated mitochondria and MAM sites (see Section 3). Furthermore, inflammation increases global DNA hypermethylation which is associated with higher mortality in CVD (Stenvinkel et al., 2007). A proposed pathway for vasoconstriction involves acid sensing channel (AISC) mediated calcium influx and activation of calcineurin and NFAT (Gonzalez Bosc et al., 2016). Myocyte stress and vasoconstriction leads to vascular impairment, pulmonary hypertension, and cardiovascular disease. AISC1 mediated events are also described in breast cancer. (Gupta et al., 2014; Gupta et al., 2016).

Osteoclast differentiation and bone remodeling is increasingly activated by extracellular acidosis-mediated RANKL-NFkB (Komarova et al., 2005), and OGR1-Calcium-NFAT (Frick et al., 2009) pathways. Hyperactivity of osteoclasts is associated with osteoporosis, sarcomas, and other skeletal diseases (Komarova et al., 2005).

### 4.5 Acidosis Facilitates Cancer Cell Survival

Acidosis activates phosphorylation of cAMP responsive element binding protein (CREB), a protein known to enhance cell proliferation and apoptosis evasion implicated in cancers (Riemann et al., 2013). A p53 mutant in adrenocortical carcinoma may be dysfunctional by acidosis induced protonation of pH sensing and regulating amino acid residues (DiGiammarino et al., 2002). Metabolic acidosis is a risk factor of breast cancer and contributes to aggressiveness of the phenotype (Park et al., 2019). Extracellular acidosis may be the cause of decreased numbers of lysosomal compartments in Human Mammary Epithelial Cells (HMECs) (Glunde et al., 2003). Decreased number and congregation of lysosomes into larger compartments is associated with more aggressive tumors (Glunde et al., 2003). Increased filipodia was found in metastatic HMECs, driven by extracellular acidosis, contributing to invasiveness (Glunde et al., 2003). Extracellular acidosis contributes to the drug resistance of tumors providing a protective mechanism by degrading the drugs before they are taken up by the cell (Avnet et al., 2016).

The Warburg effect suggests that cancer cells preferentially grow in an acidic extracellular environment. Tumor cells adapt to, and manipulate, their environment by continuously extruding acids and protons into the extracellular space (Swietach et al., 2014). Metabolic adaptation facilitates the ability of cancer cells to regain intracellular pH homeostasis, allowing survival. One suggested pathway includes MondoA, a glucose-dependent regulator of metabolism, that aids cancer cells in metabolic adaptation, apoptosis evasion, de-acidification of intracellular pH, among others (Wilde et al., 2019). A slightly more alkaline interior pH helps drive the extracellular acidification and its downstream benefits of cancer cell proliferation and energy production.

Many secondary symptoms of metabolic acidosis and chronic overnutrition contribute to risk and malignancy of cancers. Hyperglycemia increases production of ROS, where excess ROS can cause DNA damage and carcinogenesis, inflammation, and inflammation driven cancer cell survival/proliferation. High blood sugar facilitates cancer cell metabolism, generally known to be anaerobic metabolism of glucose. Decreased ability of immune cells to recognize cancer is associated with hyperglycemia (Turturro et al., 2007). Hyperinsulemia and insulin resistance are implicated in cancer cell proliferation and survival by activation of IRS-1 and RAS/MEK/ERK pathways (Law et al., 2008). While this section only provides a few examples, there are endless pathways in cancer known to be modulated by diet.

## 5 Molecular Insights Into Dietary Intervention

Nutritional components, individually or in combination, have a big impact on many of the previously described pathways in a familiar “cause-and-effect” way. Since diet provides the precursors to metabolism, it can be suggested that selective intake of dietary components can force cellular metabolic adaptation by nutrient availability, like the mechanisms exemplified in metabolic disease (Section 4.2), causing alleviation of metabolic stress and associated symptoms. To “design” an ideal diet, the therapeutic potential of all dietary components should be investigated with consideration for other factors such as variable metabolic states, digestion, absorption, microbiome interaction, and combination with other dietary and lifestyle components. Many dietary components have yet to be investigated fully, but some components known to modulate disease, pH homeostasis, and metabolism, are discussed in this section.

To neutralize acidosis and maintain arterial pH that is slightly alkaline (7.36–7.44), alkaline foods and bicarbonate supplementation are suggested. Physiological evidence supporting an alkaline shift in the diet is shown by: bone reabsorption, muscle atrophy, and excess cortisol secretion is inhibited by reducing or neutralizing dietary load (Maurer et al., 2003), alkalinizing the tumor microenvironment enhances tumor response to chemotherapy (Raghunand and Gillies, 2001), and higher levels of plasma bicarbonate is associated with lower risk for type 2 diabetes (Mandel et al., 2012). While both ways of alkalinization can relieve acidosis, digestion of more alkaline foods into their functional molecules may involve more physiological and cellular factors to holistically combat disease. Changing diet to add more fruits and vegetables reduces cardiovascular risk associated with chronic kidney disease more effectively than just sodium bicarbonate treatment (Goraya et al., 2019). Major dietary shifts will further effect the major changes in cellular metabolism required to treat or reverse the complete re-programming of cellular mechanisms caused by chronic disease. Digestion and absorption of certain foods can maintain or help regain metabolic balance at the cellular and physiological level by increased bioavailability of metabolites that can involve many pathways and effector molecules which facilitate normal metabolism and inhibit disease pathways. When those foods are also more neutral or alkaline, there is even more of an effect as dietary pH or NEAP will control acid-base balance at the molecular level.

In the gut lumen, dietary components are broken down by various digestive enzymes that operate optimally in the gut environment. Food is broken down into core metabolic building blocks-amino acids, fatty acids, simple carbohydrates (glucose), and other vitamins and minerals can also be absorbed by intestinal cells. The gut microbial community plays a vital role in human metabolism by digesting/metabolizing indigestible stuffs such as complex carbohydrates, fatty acids, and fiber. The composition of the microbiome greatly impacts many pathways, like ROS signaling associated with immune cell and metabolism regulation. Thus, it is important to consider how dietary changes will affect microbiome population and its function as it relates to the pathways discussed in previous sections and the digestion of food.

The benefits of plant-based foods in the diet are highly reviewed and widely accepted as an important part in a complete and balanced diet (Scialla and Anderson, 2013; Lynch et al., 2018). Plant material is a large source of molecules precursing bicarbonate production, resulting in a more neutral to alkaline NEAP (Strohle et al., 2011). Plant-based foods are the primary source of fiber and molecules that promote gut health, digestion, and beneficial microbial populations. Higher amounts of dietary fiber intake favored colonization with SCFA producers like *lachnospira* (Mayengbam et al., 2019). Sodium butyrate (SCFA supplement) attenuated inflammation associated with diabetes (Xu et al., 2018). SCFA’s (e.g., Butyrate) can decrease inflammation by promoting immunosuppressive cytokines (IL-10), increase ratio of regulatory Tcells, and inhibit Th17 activity. Butyrate synthesizers *rosburia* and *eubacterium* are increased in individuals with increasing mitochondrial biosynthesis and less ROS production (Ballard and Towarnicki, 2020). Colonization of the gut with SCFA producers may discourage mitochondrial dysregulation and further treat symptoms of metabolic disease. Juice from red fruits have shown increased activity of SOD and improvement for high blood pressure and cardiovascular health (Bakuradze et al., 2019). Molecules found in avocado can protect intestinal cells from absorbing lipid peroxides (Li et al., 2013).

Plant based foods have abundance of antioxidant molecules that can help reduce ROS and oxidative damage. Polyphenols (antioxidant abundant in plant foods) have low solubility into intestinal cells and so they were proposed to be good reducers of ROS that may accumulate in the blood or gut lumen. Polyphenols have been known to regulate Akt, PI3K, p53, NFkB, and positively affect oxidoreductive homeostasis (Szabo et al., 2021). More recently, contradicting evidence has discovered polyphenols generate hydrogen peroxide when circulating in the blood (Kanner, 2020). Pre-treatment, supplemental treatment, or excess consumption with polyphenols generate hydrogen peroxide (ROS) that activates Nrf2 and mediates stress adaptation and cell survival (Kanner, 2020). As with almost any dietary component, excess consumption can be a problem. Despite this newer evidence, vegetarian diets remain successful in reducing many disease symptoms, implying the generation of hydrogen peroxide by polyphenols is buffered effectively. Various combinations of phytochemicals could be responsible for this mechanism and many other benefits of plant consumption.

Vegetarian style diets can be a good way to reduce protein intake, especially of sulpher containing amino acids. Protein restriction has shown significant reduction of ammonia excretion, which is a marker for NEAP as amino acid metabolism in the kidney plays a large part in net acid production. Restricting diet from 20% protein to 6% caused alterations in the expression of ammonia creating enzymes and transporters, like PEPCK and PDG (Lee et al., 2015). This kind of diet has been shown to improve health in patients with chronic kidney diseases by decreasing glomerular pressure (Rose, 2019). Protein restriction can also improve prognosis of kidney disease aggravated by metabolic acidosis. Acidosis encourages ammoniagenesis, so reduction of the substrate is beneficial for reducing the dysfunction that follows in the kidneys.

On the other hand, high protein consumption resulted in less insulin response compared to a normal protein meal, suggesting keto diets may prevent insulin resistance and be beneficial to individuals with pre-diabetes or diabetes type 2 (Kitabchi et al., 2013). Another study showed that a high protein diet (animal and plant based alike) reduced hepatic fat deposits and inflammation in patients with type 2 diabetes (Markova et al., 2017) A high protein diet, paired with reduction in carbohydrates and slight caloric restriction, reduces total cholesterol in the blood, lipid oxidation end products, and inflammatory markers (animal protein reduces C reactive protein, plant protein reduces TNFa) (Pivovarova-Ramich et al., 2020). Most interestingly, only the animal protein group showed changes at the transcriptional level. The type of protein is also important in gut microbial health. Soy protein has been implicated in higher levels of LPS binding protein and stress response factors in serum. Soy protein had increased *ruminococcus* and lower colonization of *lactobacillus* compared to beef and chicken protein diets (Zhu Y. et al., 2017). Excess protein may increase colonization of pathogenic species of bacteria, so overconsumption of any food remains problematic. The amount of protein in a diet should be chosen with careful consideration for kidney function resulting from diabetic complications.

Reduction of glucose, energy/calorie restriction, and ketogenic diet can be beneficial in reducing visceral fat accumulation, ROS generation, lipid peroxidation, symptoms of diabetes, hypertension, and even COVID-19 complications. It has been suggested that reducing glucose and carbohydrates by ketogenic diet can reduce the amount of energy produced from aerobic glycolysis and therefore switch metabolism to be more advantageous to non-inflammatory phenotype or M2 macrophages (Sukkar and Bassetti, 2020). The fatty acids from the ketogenic diet provides substrate for oxidation in the mitochondria, which fuels oxidative metabolism that drives M2 phenotype. Glucose restriction can decrease high blood sugar in diabetic and insulin resistant patients. In kidney cells, progression of diabetic kidney disease and cardiovascular disease could be mitigated by limiting available glucose (Noce et al., 2021). Given these results, dietary carbohydrates are now being investigated as key contributors to metabolic disease, greater than fats, through their modulation of lipolysis, lipoprotein assembly and circulating levels of lipids (Hyde et al., 2019). Increase in dietary carbohydrates proportionately increases the fasting rate of carbohydrate oxidation (Miles-Chan et al., 2015).

### 5.1 The Importance of Meats in pH Homeostasis

While there is a lot of evidence and general support for increasing intake of fruit and vegetables, other dietary components have been less thoroughly investigated. Meat is nutrient dense, a good source of quality protein, Iron, Zinc, and Vitamins B12 and B6 (McNeill, 2014). Lean and unprocessed/fresh meats added into a well-balanced diet provides many nutritional and metabolic benefits, of which the mechanisms of action are just now being elucidated—challenging past paradigms. Regardless of this newer information, meat and other animal products are still recommended to be avoided by many nutrition and public health experts (WHO, CDC, among others) to reduce risk of cardiovascular, metabolic, and obesity-related disease. More in depth meta-analysis reveals that meat consumption does not increase risk of these diseases equally through the population and has variable definition and preparation (Micha et al., 2010; O’Connor et al., 2020). Meat and meat products have variable definition in type, quality, and preparation preference between geographical, cultural, and socioeconomic factors (Frank et al., 2021). The discrepancy and over generalization of the term “meat” in previous studies has only recently been identified (Binnie et al., 2014; Macho-Gonzalez et al., 2020), but has quickly created gaps in the current understanding of meat derived nutrition. For instance, red meat is proposed to have qualities that may increase polyphenol absorption into enterocytes from the gut lumen (Marin et al., 2015), which could help reduce the circulating concentration of polyphenols and resulting ROS production. However, contradictory evidence underscores the need for future studies to be more diligent and specific when investigating the kinetics of dietary components, especially in application to biological systems like human digestion and metabolism. Dietary molecules may differ in redox activity in the scope of biological systems by polarity, solubility, pH, water involvement, and metabolism. Thus, more specific, and controlled studies may be available in the future to report the benefits of meat protein sources more accurately.

Animal protein supports lean muscle mass gain and maintenance more than plant-based protein that lacks anabolic potential (Berrazaga et al., 2019). Meat protein is recommended for elderly individuals looking to maintain weight and muscle (Asp et al., 2012). It is also important to note that red meat is a good source of Iron and Zinc, heme Iron and Zinc are significantly more bioavailable in red meat than any other source (Binnie et al., 2014). Of course, excess dietary heme can cause problems, like decrease in gut microbiome alpha diversity, decrease in firmicutes, and increase in proteobacteria (esp. *enterobacteria*) species. This kind of functional group shift is associated with inflammation and cancer (Constante et al., 2017). But, in reasonable consumption, organic heme Iron is not as pro-oxidative like the inorganic ion and may not increase oxidation reactions as much as previously thought (Binnie et al., 2014). A proposed mechanism involves heme iron stabilization of Nrf2 and induction of heme oxidase 1 in the gut (Steppeler et al., 2016). Organic heme Iron promotes iron-dependent reactions, which consequently can cause ROS, but it also protects the cell from these products by signaling cellular antioxidants (Seiwert et al., 2020). Moderate ROS increase is necessary for protein synthesis/metabolism, can promote longevity and mitochondrial biosynthesis, and is normally not significant for oxidative stress (Pivovarova-Ramich et al., 2020). Animal protein diet increases ROS in the mitochondria more than plant protein, most likely due to more branched amino acids and methionine that are more susceptible to oxidation (Zhu J. et al., 2017). To confirm lack of oxidative stress, in the same study, beef protein also increased total antioxidant capacity, GSH, and SOD, promoting antioxidant activity (Zhu J. et al., 2017). Beef protein also decreased MDA (fatty acid oxidation product), which contradicts other reports that all red meat is causative of excess oxidative stress (Zhu J. et al., 2017). An explanation for low MDA may be the associated elevated levels of antioxidants, Grx1 and Trx, in the beef treatment group. These proteins are known to be upregulated in cancer to allow tumor survival and stress adaptation by reducing the excess ROS generated from increased metabolism (Zhu J. et al., 2017).

Processed or excessively fatty meat consumption results in higher levels of ROS, oxidative stress, and is more significantly associated with disease (Turesky, 2018). Saturated fats offer multiple hydrogens for excess bases to use and cause oxidation of the fatty acid carbon chain, and reduction of the base and polyunsaturated fats are most prone to oxidation. Certain processes, like curing or smoking meats, can also increase rate of meat oxidation (Zhu et al., 2014). Saturated fatty acids from the diet interact with the promoter of APOA2, thereby influencing risk of obesity depending on gene variant (Lai et al., 2018). The underlying mechanism reveals significant changes in methylation status at a particular gene location according to saturated fat intake (Lai et al., 2018). Lean meats are preferred because ROS generation decreases with decreasing fat composition and/or increasing the ratio of unsaturated fats to saturated fats may modulate redox potential. However, saturated fatty acid oxidation in meat is not as strongly associated with disease risk as high glucose, sodium, and refined carbohydrate containing diets (Baum et al., 2012). Fatty, therefore more flavorful, cuts of meat may still be allowed in moderation as part of a healthy diet with plenty of plant variety. Evidence for this is displayed by the elimination of all animal derived fats resulting in a detrimental decrease in *bacteriodales* (Gopalakrishnan et al., 2018)*.* Interestingly, fear of dietary fats (especially animal-derived fats), promoted by similar studies, has led to low fat diets that are not improving obesity and metabolic disease as much as hypothesized (Frank et al., 2016).

**FIGURE 6:**
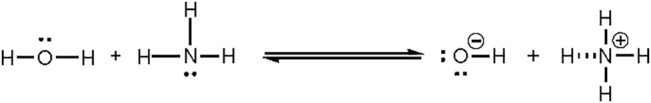
Ammonium Hydroxide equilibrium reaction. pKa of water is around 15 while the pKa of ammonia is 9.24. At pH 9–12 the reaction shifts right.

The growing meat industry is addressing public health concerns and meeting market demands by attempting to mitigate any off-target health effects through preparation and processing. More fat is being trimmed from beef cuts, ensuring at least 80% of the meat is lean (O’Connor et al., 2017). To maintain taste, optimization of flavor with limited fats is being explored through methods like; high pressure treatment above 400 MPa reduces alcohol and aldehyde formation while increasing flavor creating ketones (Zamora et al., 2020). Foods susceptible to oxidation, like meats, more likely form alkylpyrimidines, which trap reactive carbonyls (Zamora et al., 2020). It could be hypothesized that, supporting the formation of ketones and alkylpyridines with this high-pressure treatment may facilitate metabolism and reactive species scrubbing. Controlling the oxidation of meats before they are metabolized in the gut may lead to more support for the therapeutic applications of meat protein for obesity and metabolic disease. Newer experimental processing methods such as treatment of meat with selenium, a naturally occurring antioxidant, during packaging results in reduced oxidation of meat while in transit or on shelves (Korzeniowska et al., 2018). More widely used processing and packaging techniques use acidic or basic pH solutions to reduce microbes, increase tenderness, and further prepare meat for market (Vasile and Baican, 2021). Roth et al. describes injection of ammonium hydroxide (g) under high pressure for microseconds into the meat products to increase its pH (Roth, 2006). Ammonium hydroxide solution with pH above 8.0 is sufficient to raise interior pH from 6.0 up to 7.5 (Roth, 2006). This process is well known and documented to increase tenderness, water capacity, collagen solubility, protein extractability, and reduce shear force volume in meats (Hamling and Calkins, 2008). The increase in water capacity comes from combination of ammonia and hydroxide in the presence of ammonium (Figure 6). But commercial meat processes are rarely explained in the scope of human digestion and nutrition. Processed meats are also often grouped together in disease studies (O’Connor et al., 2020), so there may not be a direct explanation for some of the effects specific to each processing technique until more investigations occur, but background information could be used to extract a strong hypothesis.

**FIGURE 7:**
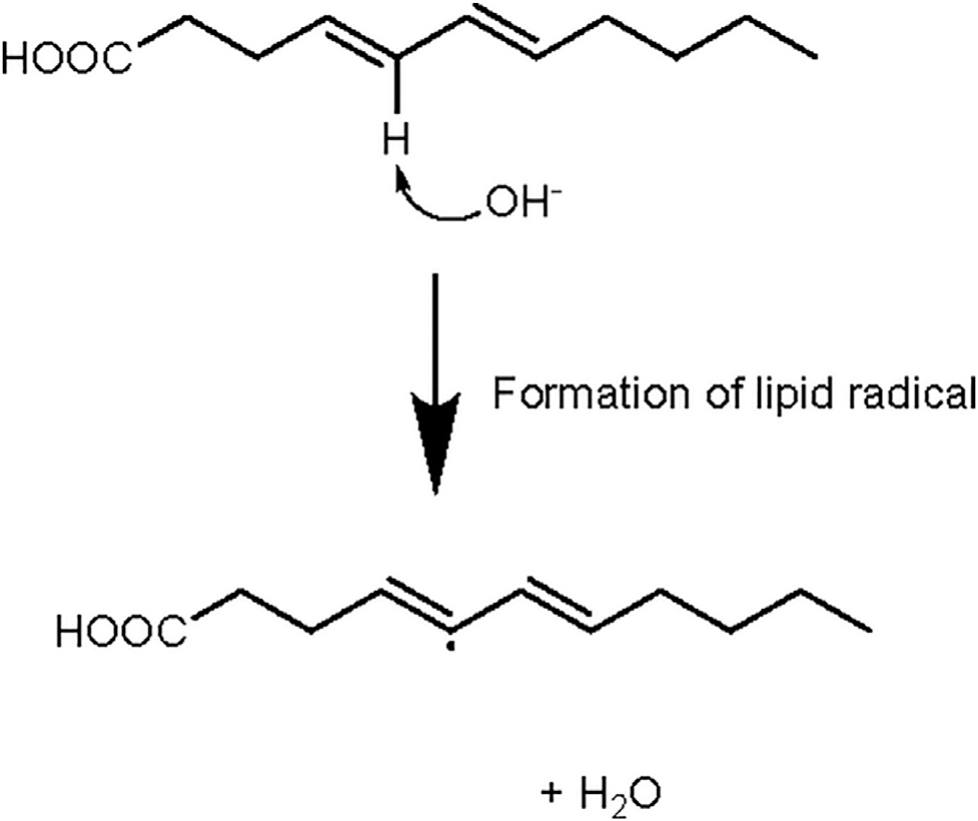
Fatty acids will create lipid peroxides or radicals and water in the presence of hydroxide. Exposure of lipids to higher pH may favor the formation of lipid radicals.

Some hypotheses on the effect of the process described by Roth et al., can be drawn from known biochemical pathways. Hydroxide is a strong nucleophile and acts as a Lewis base, while the hydroxide group donates its extra electron pair to the electrophile or proton of ammonium which is a Lewis acid. Exposing fatty acid chains and protein side groups in meat to a strong nucleophile like hydroxide (Figure 7), may allow some oxidation to occur during processing, releasing ROS outside of the body/gut, which would reduce oxidative stress and associated pathology. In contrast, slight ROS generation, that drives cellular checkpoints and metabolism are naturally induced by oxidation of meat in the gut (Pivovarova-Ramich et al., 2020). It is important to mention one study from 1921, which showed great catalytic effects on butyric acid by ammonium hydroxide (Witzemann, 1921). Ammonium hydroxide oxidized much more butyric acid than potassium hydroxide, alluding to its extreme redox ability. Witzemann (1921) describes this reaction as a model for fatty acid oxidation in acidosis of diabetic conditions and alludes to the function of ketolysis in this disease. Digestion in the gut favors ammonia + hydroxide formation because gut pH is less than the pKa of ammonia (∼9.2) and the equilibrium equation shifts right. While the hydroxyl is oxidizing lipids, excess ammonia inhibits anaerobic metabolism in pathogenic gut microbial species (Chen et al., 2016) or cancer cells. Introducing a hydroxyl scavenger through another dietary component like glucose, could potentially reduce the oxidation effect while keeping the ammonia benefits.

## 6 Conclusion

Humans are true omnivores and there may be crosstalk or synergetic events in the cell between plant and animal nutritional components that drive health and/or disease. Evidence suggests digestion of animal (oxidant) and plant (antioxidant) components together in the stomach is more beneficial to maintain redox homeostasis than meat with antioxidant supplementation (Kanner et al., 2017). Addition of garlic into ground beef reduced oxidation and improved color and microbial safety (Yang et al., 2011). Eating avocado with a hamburger reduces post meal inflammation and vasoconstriction (Li et al., 2013). To get rid of any unfavorable consequences, which result from all kinds of foods, nutritional variety and planning the right combination of oxidant/antioxidant foods may be recommended. Designing optimal redox reactions in the gut and through cell types by tailoring diet composition could be the most promising option to combat rising occurrences of all kinds of symptoms and disease. Public health, in all aspects, can benefit from continued promotion of a healthful diet, but with careful consideration for the definition of diet. With so many variables involved in the nourishment of the human body and mammalian cells, a healthful diet may be slightly different for everyone, like the differences between individuals in DNA.
